# Addressing Health Care Disparities: A Radical Perspective and Proposal

**DOI:** 10.3389/fsoc.2020.00029

**Published:** 2020-04-28

**Authors:** Donald W. Light

**Affiliations:** ^1^Professor of Sociology, Psychiatry and Comparative Health Policy Rowan University School of Osteopathic Medicine, Stratford, NJ, United States; ^2^Princeton University, Princeton, NJ, United States; ^3^New York University, New York City, NY, United States

**Keywords:** health disparities, inequality, prescription drugs, access, intellectual property, non-profit

## Abstract

This paper begins by rethinking the sociological theory that social conditions are fundamental causes of health disparities and that controlling disease ironically increases or creates them. While usually true, the radical proposal of non-profit health care and pharmaceutical development could ameliorate health disparities if a nation like Canada or a region like the EU looked to radically different but successful models such as the Drugs for Neglected Diseases *initiative*. It uses what could be called *entrepreneurial collaboration for public health markets* and inverts intellectual property to *public health IP* to maximize health gain instead of profits.

## Introduction

This essay of ideas and the sociological imagination begins by providing a new perspective on the famous studies on social conditions underlying health care disparities and on the paradox that controlling disease can create or increase such disparities. It then discusses dysfunctions of current drug development that increase disparities through high priced, patent-protected new medicines. As a radical proposal, the essay turns of the Drugs for Neglected Diseases *initiative* (DND*i*) as a working, successful model of a non-profit, virtual collaborative that researches, tests, gains approval, manufactures, and distributes clinically superior medicines at low cost and for wide access, thus reducing health care disparities. This model is illustrated by how DND*i* has developed highly effective drugs to eradicate Hepatitis C and created an example of markets to maximize public health rather than to maximize profits. Unlike other proposals to delink R&D from costs, DND*i* stands the power of patents and IP rights on their head to guarantee low prices and reduce health disparities. More work needs to be done to understand how these innovations actually work and how a theory of moral markets for public health could be developed.

Despite efforts to minimize them, health disparities plague the United States, Canada, the European Union, and regions of the world beyond. A landmark study by Link and Phelan (1995) showed that social conditions underlying health disparities are fundamental causes of diseases, not just in the US but globally. Ten years later, Phelan and Link (2005) explicated a paradox: controlling disease through costly interventions creates or increases health disparities, as people with more knowledge, money, and beneficial social connections have greater access and ability to harness medical advances and treatments than those with less. They cited evidence from the United States and Europe; but this too is a global pattern, especially for middle and lower-middle countries, where income inequalities are often even greater. For example, costly new drugs that control diseases create disparities because poorer states, subareas, and poorer individuals are less able to afford them (Iyengar et al., 2016). Costly medicines crowd out other health care services. Unequal access to needed but patented, expensive medicines exacerbates existing disparities among disadvantaged populations.

Might it be possible to control diseases without creating or increasing disparities? Could the paradox be resolved? Understanding this paradox has important implications for the kind of institutional strategies that could control disease without creating or increasing health disparities.

Let us begin by taking the fundamental or radical global perspective that all peoples have a right to effective health care and the best health possible. In fact, whole societies can organize themselves to ameliorate social conditions and maximize health and access to health care. Olafsdottir (2007) effectively illustrated this in the *Journal of Health and Social Behavior* by contrasting the social, economic, and moral conditions developed in Iceland with those in the United States. Through a constellation of acts, programs, and financing, Iceland has developed a pro-family, pro-female set of programs, while providing free, high-quality education through a university degree; universal, high-quality health care; strong pro-work provisions, and wages; robust public services and services for children and adults at all ages; and government efforts to minimize the role that markets play in providing these services. As a result, health disparities are minimized. By contrast, the United States provides only some of these services, though states vary widely along some dimensions. As a result, the percent living in poverty is much greater in the U.S., income inequality is 50% greater, female participation in the labor force is less, and women's pay is a lower percent of men's pay than in Iceland. Infant mortality is three times greater in the US, and happiness about living standards is lower. Access to effective health care is not a right in the U.S., though again, some states like California and cities like San Francisco are working hard to make it so.

Olafsdottir's study indicates that social conditions as fundamental causes of disease and health disparities can be significantly mollified and even transformed into fundamental contributors to reduced health disparities and better physical and mental health. In health care, controlling disease need not create health disparities, at least not nearly so much as is evident in a neoliberal, market-based, inegalitarian society. In theory, the paradox of creating or increasing health disparities when controlling disease need not exist. Some nations actually stand this paradox on its head: they give priority to treating those who are most disadvantaged and most ill. Challenging the “inverse care law”^1^ requires constant vigilance and re-dedication.

## Dysfunctions of Current Drug Development

Because health is a social or societal good, it is part of social justice and the meaning of a just society. A universal right to health care is also part of social and global justice, but one especially frustrated by putting the research, development, and sales of prescription drugs and vaccines into the hands of pharmaceutical companies. Over the decades, especially through the development of the pharmaceutical part of the Food and Drug Administration (FDA) in the United States, (Hilts, 2003) but also in Europe and the development of the European Medicines Agency (EMA), industry leaders have worked closely with politicians and government officials to develop a constellation of laws, practices, precedents, and institutions that minimize a focus on unmet health care needs, particularly among the poor, and maximize a focus on developing newly patented products for clinically minor variations and rare diseases (Light, 2007, 2010, 2015; Lexchin and Light, 2012). As a government-backed monopoly, patenting allows companies to charge about 50 times manufacturing costs for huge gross mark-ups on what they characterize as new, innovative medicines that they pay regulators to review (Light, 2006; Gagnon and Lexchin, 2008).

Contrary to common belief, this bald conflict of interest results in regulators approving drugs with minimal testing for clinical effectiveness or for adverse reactions (Light, 2010). This pattern lies at the center of the Risk Proliferation Syndrome. One in every four new drugs results in adverse reactions serious enough that the regulators who approved them as safe issue their most severe warning or have the drug removed from the market (Lexchin, 2012; Light et al., 2013). For priority drugs that are reviewed on an accelerated basis, that risk has risen to one in every three new drugs. Over years of independent evaluations, Prescrire found that among drugs approved, nearly twice as many are harmful enough to be regarded as not acceptable as the number found to offer a clinical advantage over existing drugs. On the effectiveness side of approvals for drugs, independent review experts conclude that 85–90% of new drugs are little or no better than existing drugs (Lexchin et al., 2003; Lexchin, 2011; Lexchin and Light, 2012). Massive marketing to physicians and patients, which companies spend more on than on research, successfully promotes these new minor variations as better and desirable (Gagnon and Lexchin, 2008). The marketing drives the Risk Proliferation Syndrome, leading to about 130,000 deaths a year in the US from prescription drugs taken properly, plus another 60,000–80,000 deaths from overdoses and misuses. The opioid crisis is a recent example of this Risk Proliferation Syndrome that has been in force for years (Light, 2010). In Europe, over 200,000 deaths from properly prescribed drugs and 20 times those numbers in hospitalizations from serious adverse reactions occur—about 4 million hospitalizations in Europe (Gøtzsche, 2013). The most frequent treatment of adverse drug reactions is to prescribe another drug, which introduced its own adverse risks.

The high costs of newer drugs, patented for profits through government-protected prices, increase inequalities of access, and health disparities and thus contribute to Phelan & Link's paradox that controlling disease may increase health disparities. Increasingly, the costs of new medicines are threatening entire health budgets and crowding out curative services (Iyengar et al., 2016). This leads to rationing, often by income. It seems time to consider a radically different way to construct research, development, manufacturing, and sales in order to reduce health disparities by addressing real health needs at low cost. Such an approach may seem utopian but is actually worked out and in operation now.

## The Drugs for Neglected Diseases *INITIATIVE* Model

Over the past 15 years, the Drugs for Neglected Diseases *initiative* (DND*i*) has developed a non-profit, public-private, virtual collaborative that is guided by principles and embodied in practices that lead to research, development, testing, and manufacturing of more effective medicines to treat patients and populations of greatest need at low cost and thus reduce health disparities (DNDi, 2019). Principles of the DND*i* model include: putting patient needs, not profits, at the heart of R&D for public health; making sure (through using patents and licensing rights to maximize public health rather profits) that better medicines are affordable and available in the communities who need them most; using open, transparent collaboration to harness the best applied science from public, academic, private sources; networking globally on “the broadest possible sharing of research knowledge and data” to facilitate scientific exchange and research capacity; and piloting new approaches to innovation for a more effective, equitable pharmacological research systems. Central to new approaches is establishing intellectual property policy around pro-access licensing terms in contractual agreements on a non-profit basis. While DND*i* has focused on neglected diseases in lower-income countries in the global South, it may serve as a model for nations or regions like the EU that wish to develop high-quality, low-cost better drugs in ways that reduce health disparities. We will start with the background of DND*i* and its development.

A seminal publication in 2001 showed that over a period of 25 years, only 1.1% of new drugs were approved specifically for neglected diseases, despite the fact that these diseases represented 12% of the global disease burden (MSF, 2001). From 2000 to 2010, that figure rose only to 4%. The report, *Fatal Imbalance*, provided the evidence needed to advocate for action and change, within and beyond the global health community. It embodied new approaches and alternative R&D models to address market and policy failures, notably by Doctors Without Borders or *Médecins Sans Frontiéres* (MSF) (MSF, 2001).

MSF used some of its Nobel Peace Prize money to cofound DND*i*, the Drugs for Neglected Disease *initiative* as a non-profit, collaborative organization, driven by patient needs and dedicated to developing more effective medicines for serious but economically neglected tropical diseases such as malaria, sleeping sickness, and Chagas disease. Its core partners consisted of five public sector institutions; one humanitarian organization, MSF; and one international research organization, the UN Development Program. The five public sector institutions were the Oswaldo Cruz Foundation from Brazil, the Indian Council for Medical Research, the Kenya Medical Research Institute, the Ministry of Health of Malaysia and France's Pasteur Institute (DNDi, 2014).

Through collaborations and partnerships with a score of pharmaceutical and biotech companies, over 50 universities and research institutes, and North-South projects, DND*i* orchestrated the development, testing, and manufacturing of six new treatments at low cost for a fraction of what pharmaceutical companies claim their R&D costs (DNDi, 2014, 2019). It has also overseen research for 12 new molecular entities (NMEs) and orchestrated 25 clinical trials for a fraction the cost reported by commercial pharmaceutical companies. These six new treatments, 12 NMEs and pivotal trials cost altogether about $200 million, about what a major company reports spending on a single phase of one drug.

This work has involved more than 125 staff in eight regional offices and over 350 collaborations in 43 countries. DND*i*'s non-profit public health projects have recruited staff and brought together research materials, and scientific know-how from nearly 20 pharmaceutical and biotechnology companies and over 50 universities and research institutes. North-South and South-South technology transfer projects and three disease-specific clinical research platforms were formed to strengthen research capacity in neglected disease-endemic countries. With its partners, DND*i* has conducted 25 clinical trials from Phase I to Phase IV implementation and pharmaco-vigilance studies, enrolling over 33,000 patients. The studies were carried out in compliance with international standards, despite often in remote and unstable areas. Suppose a constellation of states or a regional government like the EU decided to establish a non-profit, vertically and horizontally linked collaborative to undertake drug research, developing, trialing, and manufacturing. Might it cut down on patenting by largely minor innovations for profits and refocus on the public's health? Might it invert the relationship between controlling disease and health disparities? Is DNDi an example of what (Mazzucato, 2013) might call an *entrepreneurial collaboration* as an extension of the entrepreneurial state?

### An Illustration: Eradicating Hepatitis C

The growing epidemic of hepatitis C and its co-infection with HIV is growing and already causing about 400,000 deaths from HCV-related disease. Its prevalence is highly skewed toward lower -income populations and countries and disadvantaged groups within nations (DNDi, 2014; Unitaid, 2017b). Major advances known as DAAs, or direct acting antivirals, discovered largely through public funding but also by high mark-up firms, have recently revolutionized treatment. Instead of years of treatment with toxic side effects, patients are cured within a few months, with few adverse reactions. Based on patents and related rights in commercial markets, however, companies have charged up to $1,000 a pill and threatened the national budgets of even wealthy nations (Iyengar et al., 2016).

By contrast, DND*i* and its parent, Doctors Without Borders/*Médecins Sans Frontières* (MSF) started researching and testing selected combinations of newer and existing DAAs that fill genotypic gaps and are effective even in persons with HIV or HCV (Unitaid, 2017a). They are producing pan-genomic pills for patients and whole populations. They license to qualified low-cost manufacturers and construct what I think should be called “*markets for public health*,” using prices 100 times lower than in affluent, commercial markets as part of population-based campaigns (DNDi, 2018a). The nature and organization of such markets warrant further analysis.

Key to DND*i*'s methods are the roles of earmarked or special monies (Zelizer, 1994) from several partners and on an international scale. In medical and economic sociology, we need to draw on the very creative work of anthropologists like Jane Guyer to reconceive the deep inner life of currencies, especially in health care (Guyer, 2012; Wilkis, 2014). DND*i* also develops national public health markets on the “demand” side so that markets become more viable on both sides—greater risk and disease awareness, better products and more effective delivery (DNDi, 2017). How DND*i* oversees and orchestrates both the supply and demand sides of a public health market (which have very different cultural issues) warrants further study.

Since DND*i* oversees the construction of integrated, networked pre-markets for development and then markets for population-based public health, its strategies are of great interest to large states like the US or China and to regional governments like the EU. For-profit health care corporations as well as private non-profit and public institutions are involved. Theoretically, DND*i* is doing what pharmaceutical market theory says is impossible. The construction of such health care markets, their organization, ethics, and governance have wide implications for reducing health disparities elsewhere.

Markets for public health have existed for a long time; but their logic, values, organization, and structural features have not been sufficiently theorized or studied (Chorev et al., 2011). What is the coinage of the realm? What investments are being made, by whom, and for what kinds of returns? Should gain and loss measured in terms of population health gain and loss, like QALYs or DALYs? If there is a long latency period, as with reducing the risk for cervical cancer or hepatitis C or HIV, should a substantial discount rate be applied? Regardless of the answer to this question, is the goal of this kind of market increased labor force participation, productivity, and reduced health disparities, rather than profit?

The current example of Hep-C and the breakthrough of new DAA drugs has special relevance to addressing global health inequalities today (DNDi, 2019). It addresses a major problem in recent decades of new medicines that have the ability to reduce inequalities in a population's health but are priced under patent protection so that inequalities by income are exacerbated rather than reduced. To take the current example, patents and IP entanglements aside, DND*i* and MSF have shown through independent clinical trials that cost much less than big-pharma commercial trials, that *everybody* with Hep-C could afford these drugs with a cure rate of 94–96% and costs about 1% of posted prices in the United States. By contrast, monopoly pricing, and legally induced inequality have been worsened by companies proliferating minimally innovative, secondary patents, such as patenting new uses, new combinations, and a separate patent for how a given drug decomposes as it travels down the digestive tract (I-MAK, 2018). Resulting “patent thickets” stifle competition and innovation, block access and human rights, and extend monopoly rights for years.

## Patent Rights for Public Health

Theoretical and empirical studies have challenged many aspects of the standard patent claims made by companies, which hold that patents are necessary to reward the large risks and costs of research for new, “innovative” drugs with monopoly prices and defenses against competitors for a sustained period (Gøtzsche, 2013; Light et al., 2013; Gaffney et al., 2018). In response, theoretical models to delink research costs from prices have been developed (Cohen, 2002; Kapczynski et al., 2005; Outterson, 2006; Petryna, 2006; Correa, 2008, 2014; EFM't Hoen., 2009; Biehl and Petryna, 2013; Lemmens, 2013; Outterson et al., 2016; UN, 2016).

A number of complementary organizations and initiatives are addressing patent proliferation and monopoly pricing [e.g., (I-MAK, 2018)]. Nearly all of these aim to move as rapidly as possible to generic production and low costs by eliminating or circumventing patents. DND*i*, however, uses patent and licensing powers to *guarantee low prices and wide access* at low profits (DNDi, 2019). How they accomplish this is now well-known and needs further study. One might call this *patenting and IP for public health*. It uses IP and patenting rights to maximize public health gain rather than profits. This is a concept that warrants the combined interests of experts in global ethics and global health injustices. MSF and DND*i* use monopoly rights to guarantee low prices and foster competition through non-exclusive licenses, thus putting markets for public health into practice (DNDi, 2018a).

The most advanced new market being constructed to increase uptake if DAAs and reduce Hep-C is in Malaysia (DNDi, 2018b, 2019). It illustrates how important is a creative and dedicated “buyer,” like the Ministry of Health in Malaysia. Markets for public health differ radically from markets studied by economic social scientists and are designed to elevate the position of the most ill and disabled in the hierarchy of lives, rather than the most affluent and resourceful [(Fassin, 2018):124].

In conclusion, health disparities need not be exacerbated by advances in medicine. Through models like DND*i*, researchers, key actors on the demand and supply sides of health care markets, providers and suppliers can come together in joint collaborations, as is already happening in DND*i*-led markets for public health. Conceptually, one needs to broaden current theories and studies beyond commercial markets, because their foundations, cultures, concepts of “buyers” and “sellers,” and organizational features can provide insights into a more humane society, where “buyers,” “sellers,” and “consumers” work together to achieve shared, health-promoting ends. Developing the theory of markets for public health, with empirical evidence, and understanding their organizational and cultural complexity could lay the foundations for a new generation of virtual collaborations to reduce health disparities. Studies could provide the basis for how economic social scientists, welfare economists, moral philosophers, and international leaders for greater health justice conceptualize and address the inequalities of current health care markets.

## Data Availability Statement

All datasets generated and analyzed for this study are included and cited in the article/supplementary files.

## Author Contributions

The author confirms being the sole contributor of this work and has approved it for publication.

## Conflict of Interest

The author declares that the research was conducted in the absence of any commercial or financial relationships that could be construed as a potential conflict of interest.

## Footnotes

^1^The inverse care law holds that health care is inversely related to need. Those who need services least get the most, and those in greatest need get the least.
